# UNDERSTANDING THE ROLE OF SOCIAL INFRASTRUCTURE FOR OLDER ADULT ENGAGEMENT ACROSS GEOGRAPHIC CONTEXTS

**DOI:** 10.1093/geroni/igac059.1649

**Published:** 2022-12-20

**Authors:** Kenzie Latham-Mintus, Lucas Montgomery, Jeffrey Wilson

**Affiliations:** IUPUI, Indianapolis, Indiana, United States; IUPUI, Indianapolis, Indiana, United States; IUPUI, Indianapolis, Indiana, United States

## Abstract

Using semi-structured interviews of older adults, living alone or with a partner in the community, this research explores themes related to aging in place, social infrastructure, and community engagement across geographic contexts in Indiana. In particular, we are interested in understanding how older adults experience and use the existing social infrastructure in their communities and how these experiences vary across the rural-suburban-urban continuum. Additionally, we examine how social interactions supported by social infrastructure influence the maintenance of social relationships including weak social ties. Because COVID-19 has significantly changed patterns of community engagement among older adults, respondents were asked to discuss their behavior pre- and post-COVID-19. Applying a thematic analysis approach to the data, we explore the interconnections among social infrastructure, community engagement, social relationships, and geographic settings with an emphasis on older adults’ experiences and perceptions.

